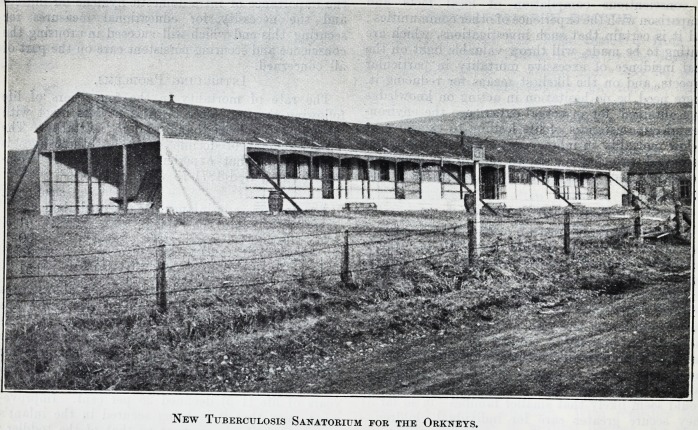# Death Rate in Childhood

**Published:** 1924-05

**Authors:** Arthur Newsholme


### DEATH-RATE IN CHILDHOOD.

### THE DECLINE BEFORE FIVE.

By SIR ARTHUR NEWSHOLME, K.C.B., M.D.

For some years past the Registrar-General, like most medical officers of health, has devoted much study and many pages of statistics to the detailed consideration of mortality in infancy and during the next four years of life. Elsewhere* I have analysed, by means of diagrams and statistics, the chief problems implied in these figures in much fuller detail than is possible here; but, as these problems have direct and immediate bearing on a large share of total public administration in this country, some of the salient points can be given. There is abundant scope for further statistical investigation in the elucidation of the various factors involved in infant and child mortality, which can be pursued by means of conscientious compilation of local facts and comparison with the experience of other communities; and it is certain that such investigations, which are waiting to be made, will throw valuable light on the local incidence of excessive mortality in particular respects, and on the likeliest means for reducing it. There need be no hesitation in acting on knowledge thus obtained, for in actual experience the environmental circumstances of life leading to excessive or to low mortality in infancy are nearly always found associated with correspondingly high or low mortality in the next four years of life, and, in fact, at higher ages also.

### Tardy Improvement.

A most remarkable feature of our national experience is that, while the general death-rate at all ages has steadily declined since the 'seventies of last century, the death-rate in infancy did not begin to show any continuous and persistent fall prior to the twentieth century. The year in which this decline began varied in different countries, and this variation forms in itself an interesting subject for investigation; but in all countries alike infantile mortality responded much more slowly to improved social, economic and sanitary conditions than the total death-rate. It would seem likely that smaller families—in so far as they secure greater care for individual children—also favour a lower infant mortality; but an investigation of the course of the national birth-rate† shows the lack of close correspondence between this and the curve of infant mortality. Thus in the year 1899 the birth-rate was 20 per cent. lower than in 1876, but in the intervening 23 years the infant death-rate showed no tendency to decline; while, on the contrary, in 1914 the birth-rate was 17 per cent. lower than in 1900, and the infant death-rate had declined 31 per cent. in 14 years. We must seek elsewhere for the chief explanation of the extraordinarily interesting fact that, whereas the English infant death-rate per 1000 births averaged 149 in 1871-80, it had become 77 in 1922 and in 1916-20 it averaged 90, and all this decline was concentrated within less than a quarter of a century.

* "Elements of Vital Statistics," new edit., 1924, chapters x., xxix., and xxx.

† See Fig. 12, *loc. cit.*, p. 115.

### Importance of Domestic Hygiene.

Many factors were at work. Improved sanitation, especially internal domestic sanitation, was an essential element in the change, and this perhaps covers most of the ground, if adequately defined. It comprised, *inter alia*, the application of antiseptic and aseptic practices in the hygiene of infancy, the application of improved methods of feeding and of general care, including exposure to air and sun; and this was rendered possible by the higher national standard of education and especially by the intensive devotion of many workers and of a majority of mothers to the special hygiene of infancy. The one great lesson of the delayed fall in infant mortality is the importance of intra-domestic hygiene for infants and the necessity for educational measures for securing this end which will succeed in arousing the conscience and securing persistent care on the part of all concerned.

### Intriguing Problems.

The rate of mortality in the four years of life following the first after birth is no less beset with intriguing problems than is infant mortality. The actual amount of decline secured at these ages is greater than that experienced for infancy. Comparing the period 1871-75 with 1911-15:—

| | | per cent. |
|---|---|---|
| The death-rate in infancy has declined about | | 29 |
| ,, | at ages 1–2 ,, ,, ,, | 61 |
| ,, | ,, 2–3 ,, ,, ,, | 50 |
| ,, | ,, 3–4 ,, ,, ,, | 42 |
| ,, | ,, 4–5 ,, ,, ,, | 51 |

But there is this difference between the above rates of decline. The fall of the infant death-rate has occurred since 1900, that at the ages 1–5 has been in progress steadily since the beginning of the 8th decade of the last century. If we compare the period 1911–14 with 1921 infant mortality declined 31 per cent., while the death-rate in the next four years of life (1–5) declined 28 per cent. Improvement, now that it is being secured in the infant's prospect of life, is as great as that of the toddler; although it will be borne in mind that improvement from a high rate of mortality is more easily secured and is more rapid than improvement of a death-rate on a lower scale.

### More Investigation Needed.

Why did the death-rate historically improve so much earlier at ages 1–5 than in infancy? The answer to this question again raises most debatable problems. Public health administration is beset with such problems. In chemistry and in physics it is often possible to arrange experiments which will enable the effect, if any, of one after another supposed factor to be determined, and will secure eventually a demonstrated proof of the truth for which search is being made. In biology, and especially in human affairs, such exact experimentation is seldom possible. When it is experienced experimentation implies a sanitary catastrophe, as in a water-borne epidemic of enteric fever, or in a limited outbreak of keratomalachia, due to a specially restricted dietary. Something may be able to be ascertained by comparison of the experience of different localities in respect of total death-rate at each year of life 1–5, and of the special causes of death at the same ages; and investigation on these lines is certain to be practically useful in many ways, and always as a pointer to special excess of mortality in respect of a particular disease or in one particular year of life. It is by such investigations in the past that driving power has been secured for some of our chief sanitary reforms. The problem is rendered the more difficult because of the intermittent prevalence of such diseases as measles and whooping cough, which take their chief toll on life at these ages. It is noteworthy that there has been improvement under both of these headings, as also in respect of bronchitis and pneumonia. The greatest improvement at these ages, as also in infancy, has been in diarrhœal mortality, and this improvement is satisfactory testimony of the steadily improving infiltration of aseptic methods of feeding into the domestic feeding of children.

### Decline in Early Tuberculosis.

But the national experience as regards tuberculosis in childhood is most striking of all. Reference to tables in the Registrar-General's Decennial Supplement and to curves on page 446 of the work already cited show that per 1,000 living the registered death-rate from total tuberculosis was higher at ages 0–5 than the total death-rate from the same disease in any part of adolescent or adult life; but that since 1910 this early tuberculosis death-rate has declined more than the tuberculosis death-rate at higher ages. It may be that a part of this change is due to change in certification of deaths in childhood; those wishing to study this point further should consult pages 17–20 of the Registrar-General's Statistical Review for 1921. The conclusion there reached, with which I agree, is that a great deal of the recorded fall is real; and we have to recognise the momentous fact that tuberculosis which in the past was an important cause of infant mortality—much more so than the official figures showed, owing to the liability to describe tuberculosis as broncho-pneumonia—is now becoming much less serious. Every infant born forms an addition to that part of the population which is extremely susceptible to the infection of tuberculosis; we know, furthermore, that bovine infection is an important but only a minor cause of infantile tuberculosis; there is reason for thinking that increasing immunity to tuberculosis is acquired as adult life approaches, if the dosage of tubercle bacilli received has been moderate or small; and we are justified, therefore, in thinking that the improvement in recent years is due in large measure to the steady diminution of opportunites for, and of dosage of, infection, both bovine and human, which have resulted from the active preventive measures adopted and the improvement in personal conduct during recent years.

New Tuberculosis Sanatorium for the Orkneys.

### A SANATORIUM IN ULTIMA THULE.

Scapa Tuberculosis Sanatorium, of which we give an illustration, is situated about a mile from Kirkwall, and faces Scapa Flow on the south, with only the width of the road between it and the sea. It is a wood and iron building, and is being reconstructed out of the officers' quarters of the Scapa Seaplane Station. There will be accommodation for a dozen patients, consisting of two four-bed wards and four single-bed wards. The staff accommodation includes five bedrooms and a nurses' dining-room. The sanatorium will possess central heating, a laundry, disinfecting hut, mortuary, etc.

## Figures and Tables

**Figure f1:**